# Supplementary material for: Keep it CooL! Results of a two-year CooL-intervention: a descriptive case series study
Source: BMC Public Health. 2024 Aug 7;24:2138. doi: 10.1186/s12889-024-19661-w (PMC11304809; doi:10.1186/s12889-024-19661-w)
Supplement: Supplementary file 1 — Supplementary Material 1 [file 12889_2024_19661_MOESM1_ESM.docx]

**S1 Table. Predictors for dropout.** Logistic regression outcomes on predictors for dropout (n=724).

| **Predictor** | **Category** | **distribution of respondents per category (%)** | **distribution of dropouts per category (%)** | **p-value** | **OR [95% CI]** |
| --- | --- | --- | --- | --- | --- |
| BMI at baseline | BMI <30* | 8.9 | 10.2 |  |  |
|  | BMI 30 - 35 | 38.9 | 44.4 | 0.926 | 0.974 [0.561; 1.693] |
|  | BMI 35 - 40 | 33.0 | 26.4 | 0.020** | 0.512 [0.290; 0.901] |
|  | BMI 40+ | 19.3 | 18.9 | 0.295 | 0.724 [0.396; 1.324] |
| Group size | Small groups (<10)* | 30.0 | 36.0 |  |  |
|  | Large groups (10+) | 70.0 | 64.0 | 0.009** | 0.629 [0.443; 0.893] |
| Educational level | Low educational level* | 27.0 | 31.1 |  |  |
|  | Intermediate educational level | 41.7 | 41.2 | 0.105 | 0.737 [0.510; 1.066] |
|  | High educational level | 31.3 | 27.7 | 0.013** | 0.606 [0.409; 0.900] |
| Gender | Male* | 28.2 | 28.7 |  |  |
|  | Female | 71,.7 | 71.3 | 0.803 | 0.959 [0.690; 1.333] |
|  | Other | 0.1 | 0.0 | 1.000 | *** |
| Age | <35* | 11.3 | 10.6 |  |  |
|  | 35 - 44 | 13.1 | 14.1 | 0.407 | 1.302 [0.698; 2.428] |
|  | 45-54 | 24.2 | 22.5 | 0.935 | 0.977 [0.561; 1.702] |
|  | 55-64 | 29.1 | 28.3 | 0.828 | 1.061 [0.619; 1.821] |
|  | 65+ | 22.3 | 24.4 | 0.296 | 1.349 [0.769; 2.366] |

* Reference category

** p<0.05

*** No calculation possible due to small number of respondents
