# Supplementary material for: Keep it CooL! Results of a two-year CooL-intervention: a descriptive case series study
Source: BMC Public Health. 2024 Aug 7;24:2138. doi: 10.1186/s12889-024-19661-w (PMC11304809; doi:10.1186/s12889-024-19661-w)
Supplement: Supplementary file 2 — Supplementary Material 2 [file 12889_2024_19661_MOESM2_ESM.docx]

**S2 Table. Changes over time CooL finishers.** Detailed overview on changes over time on participants that completely finished CooL (dataset B).

| **Category** | **Construct/ factor** | **T0 M (SD)** | **T1 M (SD)** | **T2 M (SD)** | **∆ T0T1 [95% CI]** | **Cohen’s d****  **T0T1** | **∆ T0T2 [95% CI]** | **Cohen’s d****  **T0T2** |
| --- | --- | --- | --- | --- | --- | --- | --- | --- |
| Anthro-  pometrics | Weight | 105.85 (18.01) | 101.82 (17.76) | 100.90 (18.33) | -3.42 [-4.08; -2.75]* | 0.55 | -4.67 [-5.48; -3.87]* | 0.59 |
|  | BMI | 36.27 (5.17) | 34.99 (5.32) | 34.55 (5.19) | -1.17 [-1.40; -0.94]* | 0.55 | -1.60 [-1.88; -1.32]* | 0.59 |
|  | Waist circumference | 116.43 (12.76) | 112.18 (14.37) | 111.08 (14.07) | -4.10 [-4.95; -3.25]* | 0.58 | -5.50 [-6.50; -4.50]* | 0.63 |
| Personal factors and feeling fit | Self-mastery | 2.50 (0.78) | 2.47 (0.80) | 2.39 (0.74) | -0.02 [-0.11; 0.07] | 0.02 | -0.16 [-0.21; -0.02]* | 0.15 |
|  | Perceived health | 8.98 (2.30) | 10.40 (2.08) | 10.74 (2.14) | 1.37 [1.10; 1.64]* | 0.58 | 1.82 [1.57; 2.08]* | 0.76 |
|  | Fitness (waking) | 2.47 (1.00) | 2.68 (0.86) | 2.75 (0.88) | 0.20 [0.09; 0.32]* | 0.20 | 0.29 [0.18; 0.40]* | 0.28 |
|  | Fitness (daytime) | 2.67 (0.91) | 2.70 (0.87) | 2.91 (0.84) | 0.03 [-0.10; 0.15] | 0.03 | 0.24 [0.13; 0.35]* | 0.24 |
|  | Support | 3.74 (1.03) | 3.74 (0.97) | 3.86 (0.90) | 0.07 [-0.06; 0.20] | 0.07 | 0.11 [-0.03; 0.25] | 0.10 |
|  | Influence of stress on daily functioning | 2.24 (0.98) | 2.27 (0.94) | 2.16 (0.88) | 0.05 [-0.06; 0.16] | 0.05 | -0.08 [-0.20; 0.03] | -0.08 |
| Behavioral factors | Sedentary time (least active) | 9.41 (3.93) | 8.99 (3.68) | 8.80 (3.40) | -0.23 [-0.67; 0.20] | 0.07 | -0.61 [-1.05; -0.17]* | 0.16 |
|  | Sedentary time (most active) | 6.31 (3.56) | 6.42 (3.44) | 5.90 (3.17) | -0.00 [0.42; -0.00] | 0.00 | -0.48 [-0.89; -0.07]* | 0.14 |
|  | Active minutes | 90.66 (120,72) | 100.00 (98.12) | 110.27 (112.16) | 9.82 [-2.98; 22.63] | 0.09 | 20.10 [6.71; 33.48]* | 0.18 |
|  | Sleep (summary) | 6.99 (4.34) | 6.01 (4.06) | 5.67 (3.72) | -0.97 [-1.48; -0.46]* | 0.26 | -1.22 [-1.74; -0.70]* | 0.32 |
|  | Stress (summary) | 14.65 (6.89) | 12.76 (6.55) | 12.26 (6.27) | -2.02 [-2.82; -1.23]* | 0.35 | -2.61 [-3.51; -1.72]* | 0.42 |
|  | Smoking | 0.96 (4.32) | 0.89 (3.99) | 1.04 (3.91) | -0.03 [-0.30; 0.24 | 0.01 | -0.00 [-0.38; 0.37] | 0.00 |
|  | Meal composition | 2.83 (0.99) | 3.44 (0.90) | 3.59 (0.84) | 0.62 [0.48;0.75]* | 0.57 | 0.73 [0.59;0.86]* | 0.65 |
|  | Amounts of food | 2.63 (0.92) | 3.41 (0.90) | 3.53 (0.91) | 0.78 [0.65; 0.91]* | 0.73 | 0.82 [0.69; 0.95]* | 0.87 |
|  | Attentive to consuming | 2.80 (1.13) | 3.30 (0.96) | 3.35 (0.95) | 0.47 [0.35; 0.60]* | 0.44 | 0.54 [0.42; 0.66]* | 0.49 |
|  | Alcohol | 1.58 (2.51) | 0.90 (1.47) | 0.82 (1.75) | -0.67 [-0.89; -0.45]* | 0.32 | -0.72 [-0.94; -0.49]* | 0.35 |
|  | Eating pattern*** | N/A | 4.00 [0.59] | 4.16 [0.64] | N/A | N/A | 0.15 [0.07; 0.24]* | 0.21 |

*p<0.05

** Effect size: positive values represent change in desired direction, negative values represent change in undesired direction

*** Measurement at T1 and T2: estimate of improvement in eating pattern compared to baseline, ∆T0T2 representing difference in estimate between T1 and T2.
