# Supplementary material for: Keep it CooL! Results of a two-year CooL-intervention: a descriptive case series study
Source: BMC Public Health. 2024 Aug 7;24:2138. doi: 10.1186/s12889-024-19661-w (PMC11304809; doi:10.1186/s12889-024-19661-w)
Supplement: Supplementary file 3 — Supplementary Material 3 [file 12889_2024_19661_MOESM3_ESM.docx]

**S3 Table. Subgroup educational level.** Detailed overview of the subgroup comparison on educational level (dataset A).

| **Category** | **Construct/ factor** | **LLE^1^**  **T0 M (SD)** | **IHLE^2^**  **T0 M (SD)** | **LLE**  **∆T0T2 M (SD)** | **IHLE**  **∆T0T2 M (SD)** | **P-value ∆T0T2 LLE vs IHLE** |
| --- | --- | --- | --- | --- | --- | --- |
| Anthropometrics | Weight | 106.06 (20.92) | 105.46 (17.49) | -3.20 (6.43) | -4.36 (7.78) | 0.07 |
|  | BMI | 36.60 (5.73) | 35.71 (5.08) | -1.13 (2.23) | -1.47 (2.64) | 0.15 |
|  | Waist | 118.26 (14.16) | 115.82 (12.66) | -3.58 (6.67) | -4.74 (9.31) | 0.21 |
| Personal factors & feeling fit | Self-mastery | 2.66 (0.77) | 2.49 (0.82) | -0.16 (0.70) | -0.09 (0.75) | 0.40 |
|  | Perceived health | 8.82 (2.29) | 8.96 (2.28) | 1.56 (2.18) | 1.31 (2.47) | 0.35 |
|  | Fitness (waking) | 2.41 (0.99) | 2.46 (1.02) | 0.17 (1.02) | 0.29 (0.98) | 0.21 |
|  | Fitness (daytime) | 2.57 (0.89) | 2.65 (0.94 | 0.26 (0.99) | 0.19 (0.95) | 0.46 |
|  | Support | 3.72 (1.07) | 3.72 (1.07) | 0.09 (1.00) | 0.00 (1.06) | 0.41 |
|  | Influence of stress on daily functioning | 2.11 (0.90) | 2.25 (1.00) | -0.02 (0.88) | -0.04 (1.05) | 0.84 |
| Behavioral factors | Sedentary time (least active) | 8.78 (3.59) | 9.59 (3.93) | -1.06 (3.50) | -0.47 (3.57) | 0.14 |
|  | Sedentary time (most active) | 6.31 (3.46) | 6.19 (3.52) | -0.79 (3.12) | -0.30 (3.25) | 0.18 |
|  | Active minutes | 99.63 (127.22) | 87.19 (108.83) | 49.08 (128.54) | 6.70 (106.42) | 0.00* |
|  | Sleep | 6.75 (4.35) | 6.92 (4.22) | -0.80 (3.86) | -1.14 (3.62) | 0.45 |
|  | Stress | 14.39 (6.75) | 14.37 (6.76) | -2.70 (6.90) | -1.98 (5.78) | 0.35 |
|  | Smoking | 1.25 (5.01) | 1.03 (3.97) | -0.80 (3.86) | -0.43 (3.39) | 0.25 |
|  | Meal composition | 2.82 (0.95) | 2.83 (1.00) | 0.74 (1.04) | 0.64 (1.09) | 0.42 |
|  | Amounts of food | 2.56 (0.98) | 2.66 (0.92) | 0.77 (1.16) | 0.72 (1.08) | 0.65 |
|  | Attentive to consuming | 2.96 (1.14) | 2.73 (1.11) | 0.54 (1.08) | 0.56 (1.13) | 0.83 |
|  | Alcohol intake | 1.43 (2.66) | 1.95 (2.99) | -0.89 (2.24) | -1.26 (2.69) | 0.12 |
|  | Eating pattern** | N/A | N/A | 4.08 (0.66) | 4.02 (0.71) | 0.36*** |

^1^ LLE: participants with a lower level of education (

^2^ IHLE: participants with an intermediate to higher level of education *p<0.05

*** Measurement at T1 and T2: estimate of improvement in eating pattern compared to baseline, ∆T0T2 representing difference in estimate between T1 and T2.
