# Supplementary material for: Keep it CooL! Results of a two-year CooL-intervention: a descriptive case series study
Source: BMC Public Health. 2024 Aug 7;24:2138. doi: 10.1186/s12889-024-19661-w (PMC11304809; doi:10.1186/s12889-024-19661-w)
Supplement: Supplementary file 4 — Supplementary Material 4 [file 12889_2024_19661_MOESM4_ESM.docx]

**S4 Table. Subgroup comparison by BMI category.** Detailed overview of the subgroup comparison on BMI category (dataset A)

**Table S4a.** Detailed overview of the subgroup comparison on BMI category (dataset A)

| **Category** | **Construct/ factor** | **∆T0T2**  **BMI -<30**  **M (SD)** | **∆T0T2**  **BMI 30-35**  **M (SD)** | **∆T0T2**  **BMI 35-40**  **M (SD)** | **∆T0T2**  **BMI 40+**  **M (SD)** | **ANOVA**  **p-value** |
| --- | --- | --- | --- | --- | --- | --- |
| Anthropometrics | Percentage weight loss | -1.75 (4.30) | -3.27 (6.45) | -4.46 (6.99) | -4.54 (7.73) | 0.02* |
|  | BMI | -0.49 (1.20) | -1.07 (2.10) | -1.67(2.62) | -2.02 (3.44) | 0.00* |
|  | Waist | -3.93 (4.77) | -3.36 (8.83) | -5.27 (8.67) | -4.83 (9.11) | 0.12 |
| Personal factors & feeling fit | Self-mastery | -0.15 (0.78) | -0.11 (0.71) | -0.10 (0.73) | -0.12 (0.81) | 0.99 |
|  | Perceived health | 1.31 (2.57) | 1.46 (2.32) | 1.52 (2.46) | 1.03 (2.28) | 0.48 |
|  | Fitness (waking) | 0.33 (1.08) | 0.29 (0.93) | 0.19 (1.04) | 0.26 (1.06) | 0.76 |
|  | Fitness (daytime) | 0.24 (1.00) | 0.25 (1.01) | 0.20 (0.96) | 0.15 (0.90) | 0.88 |
|  | Support | 0.04 (0.98) | -0.05 (0.97) | 0.06 (1.04) | 0.08 (1.23) | 0.81 |
|  | Influence of stress on daily functioning | -0.03 (1.14) | 0.05 (1.01) | -0.13 (1.05) | 0.00 (0.81) | 0.39 |
| Behavioral factors | Sedentary time - least active | 0.14 (4.16) | -0.77 (3.65) | -0.66 (3.50) | -0.73 (3.20) | 0.57 |
|  | Sedentary time - most active | 0.49 (3.67) | -0.57 (3.05) | -0.51 (3.39) | -0.49 (2.97) | 0.33 |
|  | Active minutes | -0.15 (134.65) | 19.22 (113.86) | 20.57 (112.51) | 19.01 (103.75) | 0.82 |
|  | Sleep | 0.09 (3.35) | -0.86 (3.73) | -1.21 (3.60) | -1.49 (3.88) | 0.31 |
|  | Stress | -1.80 (6.96) | -2.98(6.22) | -2.29 (5.99) | -1.27 (6.19) | 0.37 |
|  | Smoking | -1.33 (4.65) | -0.58 (3.98) | -0.26 (2.93) | -0.28 (4.06) | 0.25 |
|  | Meal composition | 1.03 (0.95) | 0.65 (1.06) | 0.75 (1.07) | 0.43 (1.12) | 0.03* |
|  | Amounts of food | 1.00 (0.99) | 0.66 (1.12) | 0.83 (1.08) | 0.59 (1.13) | 0.15 |
|  | Attentive to consuming | 0.89 (1.15) | 0.48 (1.09) | 0.57 (1.06) | 0.55 (1.19) | 0.24 |
|  | Alcohol consumption | -0.53 (1.74) | -1.35 (2.95) | -1.03 (2.12) | -1.10 (2.63) | 0.18 |
|  | Eating pattern ** | 4.14 (0.60) | 4.02 (0.69) | 4.00 (0.72) | 4.07 (0.71) | 0.60 |

*p<0.05

** Measurement at T1 and T2: estimate of improvement in eating pattern compared to baseline, ∆T0T2 representing difference in estimate between T1 and T2.

**Table S4b.** Details on the significant differences in BMI-subgroups.

| **Construct/item** | **Comparing subgroups** | **∆M T0T2 between subgroups** | **Explanatory summary** |
| --- | --- | --- | --- |
| BMI | BMI<30 to BMI 35-40 | 1.18* | For all three comparisons a higher BMI at baseline results in a larger decrease in BMI. |
|  | BMI <30 to BMI 40+ | 1.53* |  |
|  | BMI 30-35 to BMI 40+ | 0.96* |  |
| % Weight loss | BMI <30 to BMI 35-40 | 2.71* | For this comparison a higher BMI at baseline results in a larger percentage of weight loss |
| Meal composition | BMI <30 to BMI 40+ | 0.60* | For this comparison a higher BMI at baseline results in a smaller improvement in meal composition. |

*p<0.05
