# Supplementary material for: Keep it CooL! Results of a two-year CooL-intervention: a descriptive case series study
Source: BMC Public Health. 2024 Aug 7;24:2138. doi: 10.1186/s12889-024-19661-w (PMC11304809; doi:10.1186/s12889-024-19661-w)
Supplement: Supplementary file 5 — Supplementary Material 5 [file 12889_2024_19661_MOESM5_ESM.docx]

**S5 Table. Subgroup comparison by group size.** Detailed overview of the subgroup comparison of larger group size versus smaller group size (dataset A)

| **Category** | **Construct/ factor** | **Group size <10**  **T0 M (SD)** | **Group size 10+**  **T0 M(SD)** | **∆T0T2**  **Group size <10**  **M (SD)** | **∆T0T2**  **Group size 10+**  **M (SD)** | **P-value ∆T0T2**  **<10 versus 10+** |
| --- | --- | --- | --- | --- | --- | --- |
| Anthropometrics | Weight | 104.80 (18.43) | 106.15 (19.17) | -4.26 (6.99) | -4.23 (7.33) | 0.97 |
|  | BMI | 35.42 (4.97) | 36.38 (5.41) | -1.41 (2.28) | -1.46 (2.53) | 0.84 |
|  | Waist | 114.89 (12.41) | 116.68 (13.33) | -4.41 (7.04) | -4.36 (9.04) | 0.95 |
| Personal factors & feeling fit | Self-mastery | 2.54 (0.93) | 2.53 (0.76) | -0.01 (0.79) | -0.13 (0.73) | 0.21 |
|  | Perceived health | 8.53 (2.25) | 9.10 (2.23) | 1.66 (2.46) | 1.34 (2.31) | 0.22 |
|  | Fitness (waking) | 2.32 (1.00) | 2.50 (1.00) | 0.36 (1.04) | 0.19 (0.98) | 0.10 |
|  | Fitness (daytime) | 2.47 (0.95) | 2.67 (0.89) | 0.34 (0.98) | 0.16 (0.97) | 0.07 |
|  | Support | 3.60 (1.13) | 3.78 (1.00) | 0.01 (1.16) | 0.01 (1.02) | 1.00 |
|  | Influence of stress on daily functioning | 2.07 (0.88) | 2.22 (0.99) | 0.16 (0.96) | -0.12 (1.03) | 0.01* |
| Behavioral factors | Sedentary time (least active) | 9.51 (3.79) | 9.36 (3.88) | -0.79 (3.63) | -0.56 (3.53) | 0.55 |
|  | Sedentary time (most active) | 6.65 (3.34) | 6.24 (3.61) | -0.61 (3.14) | -0.37 (3.27) | 0.49 |
|  | Active minutes | 74.60 (82.48) | 97.90 (124.92) | 37.62 (110.60) | 8.54 (114.93) | 0.02* |
|  | Sleep | 7.49 (4.31) | 7.00 (4.32) | -1.16 (3.68) | -1.04 (3.76) | 0.81 |
|  | Stress | 14.98 (6.59) | 14.55 (6.89) | -2.23 (5.45) | -2.30 (6.40) | 0.93 |
|  | Smoking | 1.03 (4.26) | 0.95 (4.11) | -0.33 (3.15) | -0.30 (3.51) | 0.91 |
|  | Meal composition | 2.78 (0.98) | 2.88 (1.00) | 0.71 (1.06) | 0.67 (1.09) | 0.70 |
|  | Amounts of food | 2.61 (0.92) | 2.64 (0.94) | 0.76 (1.14) | 0.72 (1.07) | 0.76 |
|  | Attentive to consuming | 2.73 (1.03) | 2.85 (1.15) | 0.56 (1.12) | 0.53 (1.12) | 0.78 |
|  | Alcohol | 1.45 (2.04) | 1.75 (2.93) | -0.89 (1.86) | -0.88 (2.29) | 0.97 |
|  | Eating pattern** | N/A | N/A | 4.00 (0.62) | 4.05 (0.73) | 0.45 |

*p<0.05

**Measurement at T1 and T2: estimate of improvement in eating pattern compared to baseline, ∆T0T2 representing difference in estimate between T1 and T2.
